# *Cistus incanus* L. as an Innovative Functional Additive to Wheat Bread

**DOI:** 10.3390/foods8080349

**Published:** 2019-08-16

**Authors:** Grażyna Cacak-Pietrzak, Renata Różyło, Dariusz Dziki, Urszula Gawlik-Dziki, Alicja Sułek, Beata Biernacka

**Affiliations:** 1 Division of Cereal Technology, Faculty of Food Sciences, Warsaw University of Life Sciences, 159C Nowoursynowska St., 02-786 Warsaw, Poland; 2 Department of Food Engineering and Machines, University of Life Sciences in Lublin, 28 Głęboka St., 20-612 Lublin, Poland; 3 Department of Thermal Technology and Food Process Engineering, University of Life Sciences in Lublin, 31 Głęboka St., 20-612 Lublin, Poland; 4 Department of Biochemistry and Food Chemistry, University of Life Sciences in Lublin, 8 Skromna St., 20-704 Lublin, Poland; 5 Department of Cereal Crop Production, Institute of Soil Science and Plant Cultivation, 8 Czartoryskich St., 24-100 Puławy, Poland

**Keywords:** *Cistus incanus*, wheat, bread, baking, physical properties, antioxidants

## Abstract

*Cistus incanus* L. (CI) has been proposed as an innovative functional supplement of food products, and hence the present study aimed to evaluate the effect of the addition of dried CI on the properties of bread. Bread was prepared from white wheat flour supplemented with the addition of 1%, 2%, 3%, 4%, and 5% of ground CI. After the completion of baking process, various characteristics of the obtained bread product, such as yield, volume, porosity, acidity, color, and texture, were evaluated. In addition, total phenolic content (TPC), ABTS (2,2′-azino-bis(3-ethylbenzothiazoline-6-sulfonic acid) radical scavenging activity, chelating power (CHEL), and ability to quench OH∙ radicals were measured. The results showed that the addition of CI to bread caused a reduction in the volume of bread, but texture of the crumbs was acceptable. Acidity and moisture content of bread were found to be increased following CI enrichment. Significant changes in the ash content and the color of bread crumbs were also observed. Bread incorporated with CI was characterized by significantly higher TPC and much higher antioxidant activity, as measured by ABTS, CHEL, and OH∙ radicals, compared to control bread. Supplementation of bread with 3% CI produced a product with desirable characteristics which was also favored by consumers.

## 1. Introduction

Cistaceae is a small family of plants comprising shrubs that are commonly found in the Mediterranean climate [1,2]. The plants of *Cistus* species are used in the preparation of folk medicines, in the form of anti-diarrheal agents, to provide protection against various skin diseases, and as anti-inflammatory agents [2,3]. Küpeli and Yesilada [4] confirmed that flavonoids from *Cistus laurifolius* L. have anti-inflamatory and antinociceptive activity without causing any visible toxicity. In other studies [5] polyphenol rich extracts from *Cistus incanus* L. have a strong antiviral activity and did not exhibit apparent harming effects on cell viability, metabolism, or proliferation. Different species of *Cistus* are used as herbal infusions, food additives, or nutraceutical products [6].

*Cistus incanus* L. (CI) is included in the class of medicinal plants because it exhibits anti-inflammatory, anti-microbial, cytotoxic, and anti-ulcerogenic properties [7]. The anti-microbial potential of different extracts was tested against *Escherichia coli* and *Staphylococcus aureus* [1]. Other studies demonstrated that CI has the ability to reduce the risk of caries disease caused by *Streptococcus mutans* [8]. The anti-aflatoxigenic efficacy of the CI plant against *Aspergillus parasiticus* was also studied [9]. The tested extracts of CI [10] inhibited the growth of *A. parasiticus* and *Aspergillus carbonarius*.

The water extract of CI tea was tested to determine the concentrations of flavonols, organic acids, vitamin B, and alkaloids present in the extract [7]. Attaguile et al. [2] investigated the effects of the aqueous extracts of CI and *Cistus monspeliensis* on DNA cleavage, and their free-radical scavenging capacity was also analyzed. The impact of the extracts on lipid peroxidation in rat liver microsomes was also evaluated [11]. These products are characterized by a high content of phenolic substances and strong antioxidant activity [11,12]. Gori et al.’s study [3] aimed to characterize the major polyphenolic compounds present in a crude ethanolic leaf extract of CI.

Phenolic compounds can undergo severe changes when exposed to various processing techniques [11]. Therefore, standardizing the processing parameters is of the most importance. Domcheva et al. [13] showed that the technique and conditions adopted for extraction process have a significant impact on the yield of polyphenols and flavonoids, as well as on the antioxidant capacity of the final product. In other study [14], the influence of plant parts and particle size of CI on the extractability of phenolic compounds was demonstrated. Riehle et al. [11] investigated the influence of the brewing process involved in the preparation of CI herbal infusions on their phenolic compound profile.

In recent years, there has been a global trend toward the use of natural substances present in the food as a source of antioxidants and functional ingredients. In particular, natural antioxidants present in food have received considerable interest because of their safety profile and potential nutritional and therapeutic effects [15]. Additional ingredients are added during the bread-making process to obtain fortified bread with higher nutritive value. Bread is an important part of the diet of most people in the world; therefore, bread fortification can have a positive effect on health and the prevention of many diseases. Bread can be used as a carrier of natural source of antioxidants in food product innovation. Dried or fresh herbs can be added to the basic recipe of bread to obtain an enriched product [15,16,17,18]. For example, Zhumaliyeva et al. [19] developed a technology to prepare bakery products possessing anti-diabetic properties using a mixture of herbal supplements (rose, Jerusalem artichoke, Stevia leaf, and celery root).

Singh et al. [20] attempted to develop a functional formulation of bread by incorporating shatavari (*Asparagus racemosus* Willd.), an important medicinal plant found in India. In other study, bread products were supplemented with bioactive components such as green tea powder, herbs, and tomato paste. The bread thus obtained was called prebiotic antioxidant bread (pre-aox-bread) [21]. The effect of addition of oregano in formulation was examined in order to obtain herbal, antioxidant-enriched bread [22].

Dietary products supplemented with CI plant are increasingly offered by food manufacturers. Skąpska et al. proposed the addition of herbal CI extracts to drinks like cloudy Aronia juice [23]. In another study [24], CI leaves were proposed as an additive to pasta.

To date, no research has been done to assess the effect of the addition of dried CI on the properties of bread. Therefore, the present study aimed to determine the effect of the addition of dried and ground leaves of CI on the final quality of wheat bread and to determine the appropriate concentration of this additive that is acceptable to the consumers.

## 2. Materials and Methods

### 2.1. Raw Material

The raw materials used for the preparation of bread dough were white wheat bread flour (Polskie Młyny, Warsaw, Poland) and dried leaves of CI (Malwa Tea, Lubiszyn, Poland). The dried leaves of *Cistus* were ground into particles (measuring below 0.3 mm) in a laboratory hammer mill (POLYMIX-Micro-Hammermill MFC). Various concentrations of dried herbs (1%, 2%, 3%, 4%, and 5%) were added to the flour to obtain fortified bread. Compressed yeast (Lesaffre, Poland) and salt (Kłodawa, Poland) were purchased from the local market.

### 2.2. Chemical Composition of Raw Material

Total protein content was determined by Kjeldahl method [25] using FOSS Kjeltec 8200. Wet gluten content and gluten index were evaluated by mechanical means [26]. The falling number of flour was determined according to Hagberg-Perten method [27]. Total fat content was measured using solvent extraction method in a Soxhlet’s extractor [28]. The ash content in flour and herbs was evaluated by incinerating the sample in a muffle furnace at 900 °C for 60 min [29]. Water content was determined using drying method [30].

### 2.3. Baking Procedure of Bread

The bread dough was prepared using a straight dough method [31] and as described previously by Romankiewicz et al. [32]. The basic dough formula consisted of wheat flour (100%), water, salt (1.5% flour basis), and compressed yeast (3%). Different bread compositions were prepared using varying concentrations of CI: 1, 2, 3, 4, and 5%. Water was added to the flour according to farinograph water absorption measurements (Farinograph model 810114, Brabender, Germany) and was equal to 60.0% for the control bread and 61.5%, 62.8%, 63.7%, 64.0%, and 65.4% for bread with 1%, 2%, 3%, 4%, and 5% addition of herbs, respectively. All ingredients were mixed and fermented at 30 °C and 75% relative humidity for 60 min (with 1 min transfixion after 30 min). The dough was mixed in a laboratory mixer (SP 800A, Diskteknik, Huddinge, Sweden) and after fermentation was cut into 250 g pieces and then placed in the molds. The dough was proofed again for 60 min. The bread was baked (230 °C, 30 min) in an electric oven (Sveba Dahlen, Fristad, Sweden). After baking, loaves were cooled and packed in polyethylene bags and stored for 24 h at 20 °C.

### 2.4. Physical Properties of Bread

After the completion of baking process, the bread yield and baking losses were calculated. In addition, bread volume, crumb porosity [33], and bread acidity were estimated as previously described [34]. The loaf volume converted to 100 g of bread was determined using a 3D scanner (NextEngine, West Los Angeles, CA, USA) and calculated using a computer program (MeshLab, ISTI-CNR research center, Rome, Italy). The porosity of crumbs was measured by kneading and determining the differences in the volumes between uncompressed and compressed crumbs.

### 2.5. Color Measurements

The color parameters of CI herb and synthesized bread samples were recorded using the formula CIE-L*a*b*, where L* indicates lightness, a* redness/greenness, and b* yellowness/blueness [32,34]. The instrumental measurement of the color of dried herbs and bread products was carried out using the Minolta (CR-200, Osaka, Japan) photocolorimeter [32]. The total crumb color differences, ΔE, between bread crumbs enriched with CI and control bread sample were calculated using the formula as described previously [32,34].

### 2.6. Textural Properties of Bread Crumbs

The textural properties of the bread were analyzed using texture analyzer type TA.XT2i (Stable Microsystems, Surrey, UK). Cylindrical samples (20 mm diameter) were cut from loaf slices with a thickness of 20 mm and were compressed using a capital equipped with a 25 mm plug until a 15 mm depth at a crosshead speed of 1 mm∙s^−1^. The samples were compressed twice (curves 1 and 2) to give a two-bite TPA (Texture Profile Analysis) curve [35], from which textural parameters, such as hardness, springiness, cohesiveness, and chewiness, were calculated [32].

### 2.7. Total Phenolic Content

The TPC was determined by performing solvent extraction of bread samples. One gram of dried bread powder was extracted with 5 mL mixture of methanol and water (1:1, *v*/*v*, 30 min). Then the extracts were separated by decantation in a centrifuge (15 min). The residues were extracted again with 5 mL of methanol, and the extracts were combined and stored (−20 °C, darkness). TPC was determined according to the Folin–Ciocalteu method [36]. For each sample, 0.1 mL of extract was mixed with 0.1 mL of distilled water, 0.4 mL of Folin’s reagent (1:5 H_2_O), and later 2 mL of 10% Na_2_CO_3_ was added. The TPC was expressed in mg as gallic acid equivalents after measuring the absorbance of the mixtures in a spectrophotometer (720 nm).

### 2.8. Antioxidant Activity

Antioxidant activity of CI and bread samples was evaluated by estimating the DPPH (2,2-diphenyl-1-picrylhydrazyl) radical scavenging activity [37], the ABTS (2,2′-azino-bis(3-ethylbenzothiazoline-6-sulfonic acid) radical scavenging activity [38], the chelating power (CHEL) [39], and the ability to quench OH· radicals [40]. All anti-radical activities were expressed as EC_50_—extract concentration (mg_d.w_∙mL^−1^) that provided 50% of antioxidant activity based on a dose-dependent mode of action. EC_50_ value (mg∙mL^−1^) is the effective concentration at which the absorbance was 0.5 for RED and was obtained by interpolation from linear regression analysis. The lower EC_50_ value indicates a higher antioxidant activity.

### 2.9. Sensory Evaluation of Bread

After 24 h of baking process, sensory analysis of bread was performed under stable temperature and light conditions by a team of 67 trained panelists at Warsaw University of Life Sciences, Warsaw, Poland. Bread was cut into slices, and samples were scored using a nine-point hedonic scale according to the appearance, taste, smell, color, texture, and overall acceptability of the final product, where 1 represents “dislike extremely”, 5 indicates neither like nor dislike, and 9 represents “like extremely” [41].

### 2.10. Statistical Analysis

All tests were performed in four replicates. Statistical analyses of the obtained results were performed using Statistica software (TIBCO Software, Palo Alto, CA, USA) at a significance level of α = 0.05. The data were analyzed using analysis of variance (ANOVA), and the means were compared using the Tukey’s range test.

## 3. Results and Discussion

### 3.1. Physical Properties of Bread Fortified with Cistus incanus

Wheat flour used as a basic raw material for bread baking was characterized by a protein content of 11.3 ± 0.01%, a wet gluten content of 27.7 ± 0.14%, a gluten index of 91 ± 0.71, a falling number of 293 ± 1.41 s, a fat content of 1.8 ± 0.09%, and an ash content of 0.71 ± 0.007%. As demonstrated in an earlier study [42] and according to practical recommendations and adopted standards, wheat flour used for bread baking should have a minimum protein content of 11% and falling number must not be below 220 s. The value of gluten index indicates that the flour used in our study is characterized by good technological quality [43]. CI was characterized by a protein content of 7.9 ± 0.41, a fat content of 1.34 ± 0.71 and an ash content 5.0 ± 0.21%.

**Figure 1:**
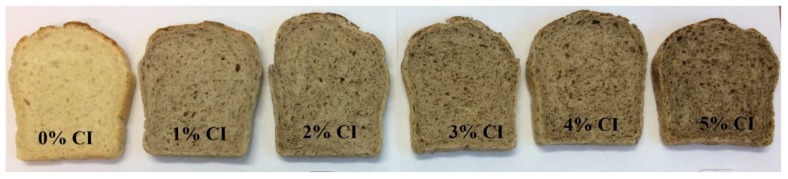
Appearance of wheat bread with different amount of *Cistus incanus* L (CI).

**Figure 2:**
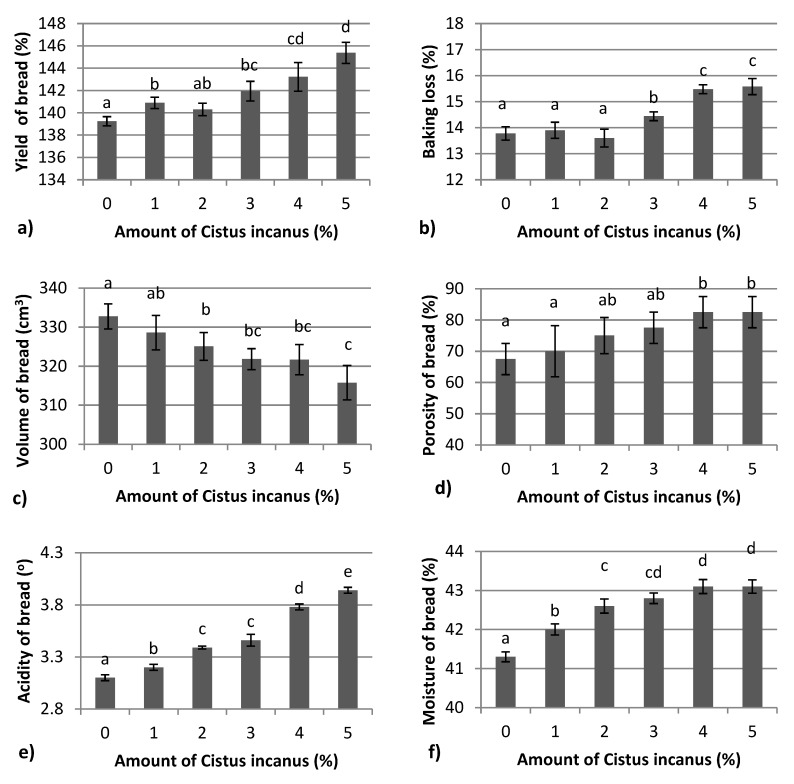
Baking properties of bread enriched with *Cistus incanus* L. additives. (**a**) yield of bread, (**b**) baking loss, (**c**) volume of bread, (**d**)porosity of bread, (**e**) acidity of bread, (**f**) moisture of bread,.

The addition of CI to bread caused noticeable changes in baking properties (Figure 1). Bread yield (Figure 2a) was found to significantly increase when 3% concentration of herbal sample was added. The bread yield depends on the amount of water absorbed by the flour during the baking process. The herbal samples of CI used in this study were characterized by 9.1 ± 0.48% water content and caused significant changes in the water absorption capacity of the bread sample, which was evident by farinograph studies which showed an increase in absorption from 60.0% to 65.4%. Other studies reported that oregano addition to bread formulation increased the water absorption capacity and dough development time, which was associated with a significant decrease in dough stability [22]. Baking loss (Figure 2b) in our studies increased significantly when 3%, 4%, and 5% concentrations of CI were used in bread formulation. The volume of bread decreased (Figure 2c), and the porosity of bread (Figure 2d) increased gradually with an increase in the proportion of these herbs from 1% to 5%. A significantly lower volume of bread was observed for the 2% concentration of CI, whereas a significantly higher porosity was characterized for bread enriched with a 4% share of herbs. A similar trend was observed in other studies, where specific volume was found to be decreased at higher concentrations of green tea powder [44]. With an increase in the level of green tea extract, no significant difference with regard to the cell diameter (porosity) was observed in the histogram [45]. The addition of green tea extract and encapsulates to bread formulation did not show any change in terms of volume, which was almost similar to control [46]. Fortification of wheat bread with CI caused significant changes in acidity (Figure 2e) and moisture content of bread (Figure 2f). As mentioned earlier, higher moisture content could be attributed to higher water absorption capacity of dough. The acidity of bread could be increased by adding different kinds of additives. One study showed that acidity of bread can be altered by the addition of crushed safflower seeds [47] or dry powder of *Chamerion angustifolium* (L.) Holub leaves [35]. Other results showed that the biologically acidified breads were characterized by increased quality including flavor, texture and shelf life [48,49].

### 3.2. Color and Texture of Bread Crumbs Modified with CI

*Cistus incanus* L. used as an additive to bread resulted in significant changes in the ash content and color of the bread (Table 1). The value of ΔE parameter of bread with a 1% addition of CI indicates a significant difference in the color of this bread from the control bread. Each subsequent amount of additive resulted in a significant increase in the ΔE parameter. The differences in color parameters of breads are caused by the pigments present in CI. In these herbs, pigments such as chlorophyll a, chlorophyl b and carrotenoids have been identified [50]. These results are consistent with previous studies, which reported that the lightness of crumb and crust decreased with increasing amounts of Moringa leaf powder, while the redness (a*) value of crumbs increased with an elevation in the concentration of this additive [50].

The addition of CI caused small changes in the textural properties of bread (Table 2). There was no significant change in the hardness of bread with the supplementation of 1%, 2%, and 3% herbs when compared to the control bread. The higher proportion, in particular 5%, of this component contributed to an increase in the hardness of the final product. The bread formulated with a 4% share of these herbs was characterized by the least springiness. The cohesiveness of bread crumbs did not show a significant change with all concentrations of herbs compared to the control bread sample. A significant increase in chewiness was observed for bread fortified with a 5% CI. Similar results were obtained for bread incorporated with black tea extract, which showed little effect on textural properties [51]. The bread sample incorporated with green tea extract and encapsulates retained their quality in terms of crumb firmness which was almost similar to control [45]. The addition of shatavari (*A. racemosus* Willd.) to the investigated bread sample affected the textural properties by increasing its crumb hardness, adhesiveness, chewiness, and cohesiveness and by decreasing the springiness [20]. Crumb hardness, cell diameter, and chewiness characteristics of whole wheat pan bread increased at higher con concentrations of green tea powder [43]. With an increase in the amount of green tea extract added, the brightness and sweetness of the bread decreased, whereas the hardness, stickiness, and astringency increased [44]. The hardness and chewiness of bread slightly decreased with the addition of 2.5% and 10% Moringa powder, whereas springiness was not found to be affected by this additive [48]. Many authors had previously established that textural attributes of bread crumbs are influenced by the nature and the amount of ingredients [52,53]. The changes related to bread crumb texture are probably a result of dilution of wheat gluten, change in the nature of starch, and enrichment of fiber content due to addition of increased amounts of CI in our study [54]. 

### 3.3. Total Phenolic Content and Antioxidant Activity of Bread Incorporated with Cistus incanus L.

The bread sample incorporated with CI was characterized by significantly higher TPC already at 1% of the amount of this additive (Table 3). A steady increase in the concentration of CI caused gradual and significant changes in the content of phenolic acids, and a more than twofold increase was observed following the addition of 5% CI. Antioxidant activity measured by ABTS, CHEL, and OH∙ was also improved by the proposed additive. The ability to quench ABTS radical, measured by EC50 value of bread, was decreased by about two times with 1% CI. The least OH^−^ radical scavenging activity and chelating power (CHEL) was observed for control bread, whereas these activities increased with increasing concentrations of CI in the formulated bread recipe. In previous scientific studies [11], thirty-two phenolic compounds among others the phenolic acids, flavan-3-ol monomers and -dimers as well as flavonol glycosides were identified in CI herbs. Mansoor et al. [55] have shown that CI contains flavanols—a complex group of polyphenols including gallocatechins responsible for anti-inflammatory properties. Regarding phenolic acids, CI samples contained especially gallic, protocatechuic and p-coumaric acids [7]. Antioxidant capacities of herbs are usually attributed to the above-mentioned phenolic compounds [11]. A few other studies confirmed the antioxidative potential of CI when included as a functional additive to pasta [24]. The antioxidative potential of bread was also improved by green tea powder, herbs, and tomato paste [21]. Bread supplemented with 1%, 2%, 3%, and 4% oregano samples showed high TPC and radical scavenging activity [22]. Black tea incorporation enhanced the antioxidant activity of Chinese steamed wheat bread [56]. Other results suggest that the addition of green tea powder at a concentration of 1.00 g/100 g of flour effectively increases the antioxidant activity of bread [43]. The TPC and antioxidant activity of bread extracts increased with the addition of Moringa leaf powder. The addition of Moringa powder already for 2.5% resulted in a large increase in the content of TPC. With regard to ABTS scavenging capacity, high activity was observed for 5.0%, 7.5%, and 10% levels. Regarding OH∙ scavenging capacity, gluten-free breads fortified with 2.5%–10% of Moringa leaf powder presented significantly higher activity compared to control bread [52].

### 3.4. Sensory Evaluation of Bread Enriched with Cistus incanus L. Herb

Sensory analysis (Table 4) showed that the addition of CI did not cause any significant change in the appearance of the bread loaves. According to the evaluators, highest scores for the appearance were obtained by breads with a 3% addition of dried herbs and the lowest scores by breads with a 5% addition, but these differences were not significantly different from the scores observed for the control bread. In terms of taste and smell, statistically significant differences were noted for bread samples fortified with 4% and 5% CI. These bread products were characterized by the lowest value compared to the control sample. Regarding the texture of crumbs, there was no statistical difference when compared to control wheat bread and bread incorporated with 1%, 2%, 3%, and 4.0% CI. Bread crumbs with 5% of herbs were characterized by the lowest score which was significantly different from the control bread. The lowest scores for overall sensory attributes were obtained by bread with 4% and 5% CI, and addition of CI up to 3% concentration resulted in a product that was rated as desirable, thus obtaining a score comparable to the control sample.

To date, no research has been conducted to determine the effect of dried CI leaves on bread characteristics. We are the first to carry out such a study with CI leaves; moreover, similar findings were reported for pasta supplemented with CI leaves [24]. However, the proportion of this additive should not exceed 3%, as it results in a change of sensory characteristics. Wheat bread can be supplemented with the same amount of Moldavian dragonhead leaves [57]. Other studies conducted on bread enriched with different herbs have shown their effect on sensory characteristics. These results suggested that oregano can be added up to 2% level to bread sample without causing any major change in baking and sensory properties and at the same time exhibiting a better shelf life [22]. The optimum acceptable level for shatavari in wheat bread was found to be 3.5% [20]. Deterioration of the organoleptic properties of bread products with herbs can be explained by the presence of essential oils that cause a specific taste and smell [58].

## 4. Conclusions

The addition of CI to bread caused significant changes in the baking properties. Bread yield and baking loss were found to be significantly increased when 3% herbs was added. The volume of bread was reduced with the increasing concentration of proposed herb. Acidity and moisture content of bread were increased when CI was used as an additive. Significant changes in ash content and color of bread crumbs were also observed. Unlike other parameters, no significant changes with regard to textural properties were noted, and only 5% addition of herbs caused significant deterioration in the texture parameters.

The bread sample incorporated with CI was characterized by significantly higher TPC and much higher antioxidant activity, as measured by ABTS, CHEL, and OH∙ radical scavenging activities, when compared to control bread sample.

Sensory analysis showed that the highest scores for overall acceptability were obtained by control bread and the lowest by bread fortified with a 5% addition of CI. Supplementation of bread with up to 3% concentration of CI produced a desirable product, which was comparable to the control sample and acceptable to the consumers. This bread was characterized by a lower volume but comparable texture and porosity and a much higher total phenolic content and antioxidant activity compared to the control bread. Bread with increased antioxidant activity should be consumed because of its role in the prevention and treatment of various chronic and degenerative human diseases.

## Figures and Tables

**Table 1:** Ash content and color parameters of *Cistus incanus* L. (CI) and bread samples supplemented with this additive.

Sample		Ash Content (%)	L*	a*	b*	ΔE
CI herbs		5.01 ^c^ ± 0.211	54.93 ^e^ ± 0.75	0.68 ^b^ ± 0.03	20.04 ^d^ ± 0.44	-
Proportion of CI added	0%	0.82 ^a^ ± 0.018	82.44 ^a^ ± 0.176	0.09 ^a^ ± 0.003	10.35 ^a^ ± 0.062	-
	1%	0.90 ^b^ ± 0.037	75.71 ^b^ ± 0.217	2.39 ^c^ ± 0.038	10.65 ^a^ ± 0.094	7.20
	2%	1.00 ^b^ ± 0.041	72.89 ^c^ ± 0.267	2.38 ^c^ ± 0.065	12.67 ^b^ ± 0.206	9.99
	3%	1.01 ^b^ ± 0.086	72.64 ^c^ ± 0.356	2.39 ^c^ ± 0.037	12.69 ^b^ ± 0.235	10.12
	4%	1.06 ^b^ ± 0.027	70.90 ^d^ ± 0.206	2.91 ^d^ ± 0.063	14.45 ^c^ ± 0.184	12.21
	5%	1.10 ^b^ ± 0.064	69.98 ^d^ ± 0.330	2.88 ^d^ ± 0.065	14.59 ^c^ ± 0.227	13.09

Note: Means with different letter in the same column are significantly different (α = 0.05).

**Table 2:** Texture parameters of bread samples enriched with *Cistus incanus* L. (CI).

Sample		Hardness (N)	Springiness (-)	Cohesiveness (-)	Chewiness (N)
Proportion of CI added	0%	12.9 ^a^ ± 0.59	0.91 ^a^ ± 0.031	0.61 ^a^ ± 0.041	7.2 ^ab^ ± 0.53
	1%	12.7 ^a^ ± 0.78	0.88 ^ab^ ± 0.037	0.57 ^a^ ± 0.038	6.4 ^b^ ± 0.41
	2%	12.8 ^a^ ± 0.68	0.89 ^a^ ± 0.028	0.56 ^a^ ± 0.031	6.5 ^b^ ± 0.36
	3%	12.9 ^a^ ± 0.94	0.88 ^ab^ ± 0.045	0.55 ^a^ ± 0.018	6.6 ^ab^ ± 0.40
	4%	13.7 ^ab^ ± 0.63	0.86 ^b^ ± 0.019	0.56 ^a^ ± 0.011	6.7 ^ab^ ± 0.35
	5%	14.3 ^b^ ± 0.46	0.89 ^a^ ± 0.026	0.57 ^a^ ± 0.026	7.4 ^a^ ± 0.41

Note: Means with different letter in the same column are significantly different (α = 0.05).

**Table 3:** Total phenolic content and antioxidant activity of bread supplemented with *Cistus incanus* L. (CI).

Sample		(mg _GAE_∙g _d.w._^−1^)	EC_50_ (mg _d.w._∙mL^−1^)		
		TPC	ABTS	CHEL	OH∙
Amount of CI added	0%	4.8 ^a^ ± 0.06	156.9 ^a^ ± 9.83	65.3 ^a^ ± 0.36	39.9 ^a^ ± 2.11
	1%	5.6 ^b^ ± 0.06	77.5 ^b^ ± 0.92	43.8 ^b^ ± 2.35	35.0 ^b^ ± 1.10
	2%	6.7 ^c^ ± 0.10	48.6 ^cd^ ± 1.25	40.7 ^bc^ ± 0.58	26.9 ^c^ ± 1.21
	3%	7.9 ^d^ ± 0.25	38.3 ^de^ ± 1.55	38.1 ^cd^ ± 0.32	25.7 ^c^ ± 0.83
	4%	8.3 ^e^ ± 0.12	33.1 ^e^ ± 1.15	36.7 ^d^ ± 0.56	24.8 ^cd^ ± 0.95
	5%	10.1 ^f^ ± 0.06	24.8 ^e^ ± 0.70	33.9 ^e^ ± 0.83	22.5 ^d^ ± 0.89

Notes: TPC—total phenolic content; ABTS—ability to quench ABTS radicals; CHEL—chelating power; OH—ability to quench OH∙ radicals. Values in the same column not sharing same letters are significantly different (α = 0.05).

**Table 4:** Sensory analysis of wheat bread enriched with *Cistus incanus* L. (CI).

Amount of CI Added	Sensory Attributes				
	Appearance	Taste	Aroma	Texture	Overall Acceptability
0%	6.3 ^a^ ± 0.62	6.7 ^a^ ± 0.76	6.8^a^ ± 0.93	7.0 ^a^ ± 0.87	6.7 ^a^ ± 0.84
1%	6.4 ^a^ ± 0.87	6.6 ^a^ ± 0.79	6.9^a^ ± 0.85	6.6 ^ab^ ± 0.73	6.5 ^a^ ± 0.73
2%	6.7 ^a^ ± 0.91	6.1^ab^ ± 0.59	6.5 ^ab^ ± 0.74	6.4 ^ab^ ± 0.81	6.5 ^a^ ± 0.82
3%	6.8 ^a^ ± 0.83	5.6 ^ab^ ± 0.63	5.8 ^ab^ ± 0.62	6.3 ^ab^ ± 0.75	6.4 ^a^ ± 0.81
4%	6.0 ^a^ ± 0.59	5.1 ^b^ ± 0.71	5.3 ^b^ ± 0.63	6.0 ^ab^ ± 0.79	5.2 ^b^ ± 0.37
5%	5.5 ^a^ ± 0.64	5.0 ^b^ ± 0.65	5.2 ^b^ ± 0.59	4.9 ^b^ ± 0.56	5.1 ^b^ ± 0.44

Note: Values followed by the same letter in the same column are not significantly different (*α* = 0.05).
